# Nationwide Case-Control Study Examining the Association between Tamoxifen Use and Alzheimer’s Disease in Aged Women with Breast Cancer in Taiwan

**DOI:** 10.3389/fphar.2017.00612

**Published:** 2017-09-05

**Authors:** Kuan-Fu Liao, Cheng-Li Lin, Shih-Wei Lai

**Affiliations:** ^1^College of Medicine, Tzu Chi University Hualien, Taiwan; ^2^Department of Internal Medicine, Taichung Tzu Chi General Hospital Taichung, Taiwan; ^3^Graduate Institute of Integrated Medicine, China Medical University Taichung, Taiwan; ^4^College of Medicine, China Medical University Taichung, Taiwan; ^5^Management Office for Health Data, China Medical University Hospital Taichung, Taiwan; ^6^Department of Family Medicine, China Medical University Hospital Taichung, Taiwan

**Keywords:** Alzheimer’s disease, breast cancer, woman, Taiwan National Health Insurance Program, tamoxifen

## Abstract

**Background and Objectives:** Little is known about the association between tamoxifen use and Alzheimer’s disease in women with breast cancer. The study aimed to explore the association between tamoxifen use and Alzheimer’s disease in aged women with breast cancer in Taiwan.

**Methods**: We conducted a retrospective nationwide case-control study using the database of the Taiwan National Health Insurance Program. Totally, 173 female subjects with breast cancer aged ≥ 65 years with newly diagnosed Alzheimer’s disease from 2000 to 2011 were identified as the cases. Additionally, 684 female subjects with breast cancer aged ≥ 65 years without any type of dementia were selected as the matched controls. The cases and the matched controls were matched with age and comorbidities. Ever use of tamoxifen was defined as subjects who had at least a prescription for tamoxifen before the index date. Never use of tamoxifen was defined as subjects who never had a prescription for tamoxifen before the index date. We used the logistic regression model to calculate the odds ratio (OR) and 95% confidence interval (CI) of Alzheimer’s disease associated with tamoxifen use.

**Results**: The OR of Alzheimer’s disease was 3.09 for subjects with ever use of tamoxifen (95% CI 2.10, 4.55), compared with never use. The OR of Alzheimer’s disease was 1.23 for subjects with increasing cumulative duration of tamoxifen use for every 1 year (95% CI 1.13, 1.34), compared with never use.

**Conclusion:** The increased odds of Alzheimer’s disease associated with tamoxifen use may be due to the survival effect, not the toxic effect. That is, the longer the tamoxifen use, the longer the patients survive, and the greater the likelihood that she may have a chance to develop Alzheimer’s disease.

## Introduction

Tamoxifen has been widely used as an adjuvant therapy for estrogen receptor-positive breast cancer with good efficacy (Davies et al., 2013; Smith, 2014). The side effects of tamoxifen use include vasomotor and gynecologic symptoms, such as hot flashes, night sweats, and vaginal dryness, and other symptoms, such as weight gain, insomnia, and joint aches (Day et al., 1999; Garreau et al., 2006; Smith, 2014). Recent *in vitro* studies have shown that tamoxifen can protect neuronal cells against oxidative stress-mediated mitochondrial dysfunction (Moreira et al., 2005; Wakade et al., 2008). That is, tamoxifen use may have a potential role for the neurodegenerative disorders (Arevalo et al., 2011, 2012).

Alzheimer’s disease is one of the most commonest neurodegenerative disorders. Some evidence has shown that mitochondrial dysfunction may play a role on the pathogenesis of Alzheimer’s disease (Sompol et al., 2008; Cadonic et al., 2016). To date, no epidemiological study explores the association between tamoxifen use and Alzheimer’s disease in women with breast cancer. Given female breast cancer was the fourth cause of cancer death in Taiwan in 2016 (Ministry of Health and Welfare, 2017a) we conducted a retrospective nationwide case-control study to explore the association between tamoxifen use and Alzheimer’s disease in aged women with breast cancer in Taiwan.

## Materials and Methods

### Data Source

Taiwan is an independent country with more than 23 million people (Huang and Chang, 2016; Maa and Leu, 2016; Ooi, 2016; Yu et al., 2016; Chen et al., 2017; Lee et al., 2017). We conducted a retrospective nationwide case-control study to analyze the database of the Taiwan National Health Insurance Program. This insurance program began in March 1995 and the enrollment rate was over 99.6% of 23 million people living in Taiwan in 2015 (Ministry of Health and Welfare, 2017b). The details of the program can be found in previous studies (Lai et al., 2010; Chen et al., 2016; Tsai et al., 2016; Liao et al., 2017a,b). The study was approved by the Research Ethics Committee of China Medical University and Hospital in Taiwan (CMUH-104-REC2-115).

### Sampled Subjects

Totally, 173 Female subjects with breast cancer aged 65 years and older who were newly diagnosed with Alzheimer’s disease (ICD-9 code 331.0) from 2000 to 2011 were identified as the cases. The date of a subject being diagnosed with Alzheimer’s disease was defined as the index date. Additionally, 684 female subjects with breast cancer aged 65 years and older who never had any type of dementia were selected from the same database as the matched controls. The cases and the matched controls were matched with age (every 5-year interval), comorbidities, and the year of index date.

### Comorbidities

Comorbidities which could be potentially related to Alzheimer’s disease before the index date were included as follows: alcohol-related disease, cerebrovascular disease, chronic kidney disease, chronic obstructive pulmonary disease, diabetes mellitus, hyperlipidemia, and hypertension. Based on the ICD-9 codes, the diagnosis accuracy of comorbidities has been well-evaluated in previous studies (Lai et al., 2013, 2017a,b; Hung et al., 2015; Shen et al., 2016).

### Measurements of Tamoxifen Use and Aromatase Inhibitors Use

Prescription history of tamoxifen and aromatase inhibitors was included in the study. The measurements of medications use were adapted from previous studies (Lai et al., 2015, 2016; Cheng et al., 2017). Ever use of medications was defined as a subject why had at least a prescription for medications studied before the index date. Never use of medications was defined as a subject who never had a prescription for medications studied before the index date.

### Statistical Analysis

We compared the distributions of the demographic status, tamoxifen use, aromatase inhibitors use, and comorbidities between the cases and the matched controls using the Chi-square test for categorized variables. The *t*-test was used to examine the differences of mean age and mean duration of exposure to tamoxifen between the cases and the matched controls. Only a univariable logistic regression model was used due to no other variable being significantly related to Alzheimer’s disease. We further conducted an analysis about the duration-dependent effect of tamoxifen use on the risk of Alzheimer’s disease. All analyses were performed by SAS statistical software (version 9.2; SAS Institute, Inc., Cary, NC, United States). The results were considered statistically significant when two-tailed *P*-values were less than 0.05.

## Results

### Characteristics of the Study Population

**Table 1** discloses the characteristics of the study population. We identified 173 cases with newly diagnosed Alzheimer’s disease in 2000–2011 and 684 matched controls, with a similar distribution of age. The mean ages (standard deviation) were 80.2 (5.61) years in cases and 80.0 (5.69) years in matched controls, without statistical significance (*t*-test, *P* = 0.19). The mean durations of exposure of tamoxifen (standard deviation) were 2.55 (2.00) years in cases and 2.28 (1.80) years in matched controls, without statistical significance (*t*-test, *P* = 0.15). The cases with Alzheimer’s disease were more likely to have a higher proportion of ever use of tamoxifen than the matched controls (77.5% vs. 52.6%, Chi-square test, *P* < 0.001). There was no significant difference in the distributions of ever use of aromatase inhibitors and comorbidities between the cases and the matched controls (Chi-square test, *P* > 0.05 for all).

**Table 1:** Characteristics of cases with Alzheimer’s disease and matched controls.

	Cases with Alzheimer’s disease *N* = 173		Matched controls *N* = 684		
Variable	*n*	(%)	*n*	(%)	*P*-value^∗^
Age group (years)					0.99
65–74	32	(18.5)	128	(18.7)	
75–84	110	(63.6)	436	(63.7)	
≥85	31	(17.9	120	(17.5)	
Age (years), mean (standard deviation)^†^	80.2	(5.61)	80.0	(5.69)	0.19
Duration of exposure to tamoxifen (years), mean (standard deviation)^†^	2.55	(2.00)	2.28	(1.80)	0.15
Ever use of tamoxifen	134	(77.5)	360	(52.6)	<0.001
Ever use of aromatase inhibitors	30	(17.3)	88	(12.9)	0.13
Comorbidities					
Alcohol-related disease	1	(0.58)	3	(0.44)	0.81
Cerebrovascular disease	24	(13.9)	89	(13.0)	0.76
Chronic kidney disease	12	(6.94)	45	(6.58)	0.87
Chronic obstructive pulmonary disease	48	(27.8)	189	(27.6)	0.98
Diabetes mellitus	66	(38.2)	260	(38.0)	0.97
Hyperlipidemia	78	(45.1)	305	(44.6)	0.91
Hypertension	143	(82.7)	564	(82.5)	0.95

*Data are presented as the number of subjects in each group, with percentages given in parentheses, or mean with standard deviation given in parentheses. ^∗^Chi-square test, and ^†^*t*-test comparing cases with Alzheimer’s disease and matched controls*.

### Risk of Alzheimer’s Disease Associated with Tamoxifen Use, Aromatase Inhibitors Use, and Comorbidities

**Table 2** discloses the risk of Alzheimer’s disease associated with tamoxifen use, aromatase inhibitors use, and comorbidities. The univariable logistic regression model disclosed that the OR of Alzheimer’s disease was 3.09 for subjects with ever use of tamoxifen (95% CI 2.10, 4.55), compared with never use.

**Table 2:** Odds ratio and 95% confidence interval of Alzheimer’s disease associated with tamoxifen use, aromatase inhibitors use, and comorbidities.

Variable	OR^†^	(95%CI)
Age (per 1 year)	1.02	(0.99, 1.05)
Tamoxifen (never use as a reference)		
Ever use	3.09	(2.10, 4.55)
Aromatase inhibitors (never use as a reference)		
Ever use	1.42	(0.90, 2.24)
Comorbidities (yes vs. no)		
Alcohol-related disease	1.32	(0.14, 12.8)
Cerebrovascular disease	1.08	(0.66, 1.75)
Chronic kidney disease	1.06	(0.55, 2.05)
Chronic obstructive pulmonary disease	1.01	(0.69, 1.46)
Diabetes mellitus	1.01	(0.71, 1.42)
Hyperlipidemia	1.02	(0.73, 1.43)
Hypertension	1.01	(0.65, 1.58)

^†^*Because no other variable was significantly related to Alzheimer’s disease in a univariable logistic regression model, we did not perform a multivariable logistic regression model*.

### Risk of Alzheimer’s Disease Associated with Cumulative Duration of Tamoxifen Use

**Table 3** discloses a sub-analysis on the risk of Alzheimer’s disease associated with cumulative duration of tamoxifen use. The OR of Alzheimer’s disease was 1.23 for subjects with increasing cumulative duration of tamoxifen use for every 1 year (95% CI 1.13, 1.34), compared with never use.

**Table 3:** Cumulative duration of tamoxifen use and the risk of Alzheimer’s disease.

Variable	Case number /control number	OR^†^	(95% CI)
Never use of tamoxifen as a reference	39/324	1.00	(Reference)
Cumulative duration of tamoxifen use (increase in duration per 1 year)	134/360	1.23	(1.13, 1.34)

^†^*Because no other variable was significantly related to Alzheimer’s disease in a univariable logistic regression model, we did not perform a multivariable logistic regression model*.

## Discussion

In this retrospective nationwide case-control study, we noted that tamoxifen use was associated with 3.09-fold increased odds of Alzheimer’s disease. We noted that the odds would be increased as the cumulative duration of tamoxifen use was increased (**Table 3**). It indicates that there could be a duration-dependent effect of tamoxifen use on the risk of Alzheimer’s disease. Our findings are contrary to *in vitro* studies showing that tamoxifen may have a protective role on the neurodegenerative disorders mediated by reducing oxidative stress-related mitochondrial dysfunction (Moreira et al., 2005; Wakade et al., 2008). There seems to be a discrepancy between the epidemiological and *in vitro* studies. We reviewed the references to explain the discrepancy. The prevalence of Alzheimer’s disease is highly associated with increasing age (Qiu et al., 2009; Sosa-Ortiz et al., 2012). That is, the older the patients, the greater the likelihood that he or she would have an Alzheimer’s disease. In addition, long-term continuing use of tamoxifen for 10 years could reduce recurrence and mortality of breast cancer, compared with only 5 years of tamoxifen use (Davies et al., 2013; Smith, 2014). That is, long-term of tamoxifen use is associated with improved survival. Thus, patients who use tamoxifen longer may survive longer. They are more likely to develop Alzheimer’s disease later. That is, the longer the tamoxifen use, the longer the patients survive, and the greater the likelihood that she may have a chance to develop Alzheimer’s disease. Therefore, we think that the increased odds of Alzheimer’s disease associated with tamoxifen use may be due to the survival effect, not the toxic effect.

## Limitation and Strength

There are several limitations in this study. First, due to the limitation of a case-control study, a causal relationship between tamoxifen use and Alzheimer’s disease cannot be established. Second, our sample size is small. We cannot perform a cohort study. A cohort design does not have the drawback highlighted above for case-control studies. A prospective cohort study with a large sample size is needed for a more detailed analysis of this issue. Third, this study relied on diagnostic codes to establish Alzheimer’s disease and other comorbidities, which inevitably introduces some misclassification of cases. In methods section, we mentioned that the cases and the matched controls were matched with comorbidities. It is less likely that misclassification would be more common in tamoxifen users than non-users. Therefore, the results may not be substantially affected. Fourth, this is a retrospective case-control study. We retrospectively explored whether tamoxifen use has an association with the development of Alzheimer’s disease. That is, tamoxifen use precedes the diagnosis of Alzheimer’s disease. As a general rule, no anti-Alzheimer’s disease drugs can be used before the confirmed diagnosis of Alzheimer’s disease is made. Therefore, it is not reasonable to explore any pharmacological interaction between tamoxifen and anti-Alzheimer’s disease drugs.

There are several strengths in this study. This is the first nationwide case-control study to explore the association between tamoxifen use and Alzheimer’s disease in aged women with breast cancer. The methodology is appropriate. The results are clearly presented. It appears to be informative and influential on its area of research.

## Conclusion

The increased odds of Alzheimer’s disease associated with tamoxifen use may be due to the survival effect, not the toxic effect. Though tamoxifen use may increase survival, it lets women to be at risk of developing Alzheimer’s disease in later life.

## Author Contributions

K-FL planned and conducted this study. He participated in the data interpretation, and he revised the article. C-LL conducted the data analysis and revised the article. S-WL planned and conducted this study. He contributed to the conception of the article, initiated the draft of the article, and revised the article.

## Conflict of Interest Statement

The authors declare that the research was conducted in the absence of any commercial or financial relationships that could be construed as a potential conflict of interest.
